# ExpressionData - A public resource of high quality curated datasets representing gene expression across anatomy, development and experimental conditions

**DOI:** 10.1186/1756-0381-7-18

**Published:** 2014-08-31

**Authors:** Philip Zimmermann, Stefan Bleuler, Oliver Laule, Florian Martin, Nikolai V Ivanov, Prisca Campanoni, Karen Oishi, Nicolas Lugon-Moulin, Markus Wyss, Tomas Hruz, Wilhelm Gruissem

**Affiliations:** 1 Department of Biology, ETH Zurich, 8092 Zurich, Switzerland; 2 Institute of Theoretical Computer Science, ETH Zurich, 8092 Zurich, Switzerland; 3 NEBION AG, Hohlstrasse 515, 8048 Zurich, Switzerland; 4 Philip Morris International R&D, Quai Jeanrenaud 5, 2003 Neuchatel, Switzerland

## Abstract

Reference datasets are often used to compare, interpret or validate experimental data and analytical methods. In the field of gene expression, several reference datasets have been published. Typically, they consist of individual baseline or spike-in experiments carried out in a single laboratory and representing a particular set of conditions.

Here, we describe a new type of standardized datasets representative for the spatial and temporal dimensions of gene expression. They result from integrating expression data from a large number of globally normalized and quality controlled public experiments. Expression data is aggregated by anatomical part or stage of development to yield a representative transcriptome for each category. For example, we created a genome-wide expression dataset representing the FDA tissue panel across 35 tissue types. The proposed datasets were created for human and several model organisms and are publicly available at http://www.expressiondata.org.

## Background

Baseline or reference data are important for the analysis and interpretation of experiments and for testing methods and algorithms. Two main types of baseline data exist: a) measurements that serve as negative controls within a given experiment, and b) experiments that were carried out for the purpose of creating a collection of default states. In the first type, data from control samples (typically unperturbed samples) are necessary to test the effect of individual factors in an experiment or to calibrate a technology or treatment. In the second type, baseline datasets typically aim at compiling a large collection of data points representative for development, anatomy, cancer or response to a particular condition. The main objective of the second type is to provide comparative value for improved interpretation or for verification. In the present work, we consider the second type of reference data.

The nature of what are considered to be appropriate baseline conditions depends on community standards and on experimental design, but frequently they target unperturbed, healthy samples from a “wild-type” genetic background, or they represent a collection of perturbed states as reference for one’s own data. Several distinct types of baseline gene expression data exist: tissue, cancer or development profiles (usually absolute expression values), perturbations and diseases (relative values) and time courses and dose responses (absolute or relative values), or a combination of these spatial, temporal and response profiles. Examples of baseline data sets that have been published include the GNF human tissue panel [1], The Cancer Genome Atlas [2], the rat liver and kidney HESI baseline dataset [3], the NOWAC Postgenome Study [4], and the Arabidopsis AtGenExpress datasets for abiotic stress [5], plant development [6], or hormonal and chemical responses [7]. New technological platforms are also frequently assessed by generating data from control samples, resulting in baseline experimental datasets that can subsequently be used as a reference on these platforms. For example, several normalization methods for Affymetrix expression arrays have been benchmarked using the Latin square spike-in data from Affymetrix [8]. Further examples of spike-in datasets are the Golden Spike [9], Platinum Spike [10] and Agilent Spike [11] experiments to assess single channel or dual channel microarrays, and a spike-in dataset used to map the mammalian transcriptome using high-throughput RNA sequencing [12].

Most publicly available datasets originate from a single experiment with few independent biological replicates and performed in a particular experimental setting. The expression profiles of these samples therefore represent gene expression in that particular context, but it is not a priori clear whether these results are generally reproducible in other contexts. Furthermore, it is not immediately visible whether similar results have already been found previously or if they are novel. To verify these two questions requires the availability of comparable experiments. Comparing one’s own expression profiles with reference datasets composed of a variety of different experimental conditions allows interpreting similarities globally or at the level of individual gene signatures. It is clear that the composition and robustness of the reference datasets used will have a major impact on the outcome of such comparisons. It is therefore essential to create robust reference datasets containing representative expression values for individual biological contexts. Robustness and improved statistical power can be achieved through intensity-level integration of microarray data [13]. The quality of these profiles also depends on the quality and granularity of the sample annotations. Here, we present a collection of reference datasets based on average expression values generated from many samples originating from a similar biological context. The annotations of each sample were manually verified and their profiles were compared to other samples having the same annotations. The reasoning for this approach is that the expression of a set of genes in a specifically defined condition is reproducible and, therefore, similar data sets can be combined to create a representative profile. This concept, called meta-profiling, has been introduced in GENEVESTIGATOR and has proven to be highly useful [14]. The approach works particularly well for tissue types and cancers, since they are the main determinant of transcript population [15]. It allows creating rich and robust datasets from the bulk of research data that is publicly available for various applications, in particular for confirmation, classification or interpretation of one’s own experimental results.

## Construction and content

### Data preparation

**Figure 1:**
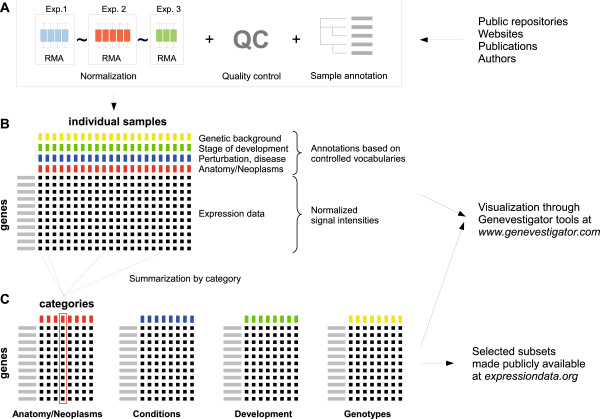
**Data transformation process from public repositories to unified data queried by GENEVESTIGATOR or to reference datasets available at **http://www. expressiondata.org**.A**. Manual curation of public data, including sample annotation using ontologies, statistical quality control of samples and experiments, and data normalization. **B**. Storage of high quality standardized expression experiments in the GENEVESTIGATOR database. **C**. Aggregation of expression data according to categories of anatomical parts, cell lines, neoplasms, perturbations (including diseases, drugs and genotypes) and stages of development. While all datasets are available through GENEVESTIGATOR, selected compilated reference datasets such as for anatomy or development are made publicly available through http://www. expressiondata.org.

The new resource, called ExpressionData and available at http://www.expressiondata.org, provides reference datasets for human and several model organisms (mouse, Drosophila, Arabidopsis, rice, and yeast), for different technological platforms, and for a variety of biological dimensions (tissue types, developmental stages, or time courses). The resource primarily contains subsets of data that were generated from the GENEVESTIGATOR expression compendium [14]. In brief, GENEVESTIGATOR is a high quality, manually curated and well annotated compendium of expression data collected from a variety of public repositories, including Gene Expression Omnibus [16] and ArrayExpress [17]. All samples were annotated using controlled vocabularies from ontologies for anatomical parts, stages of development, perturbations (diseases, chemicals, hormones, etc), genotypes (genetic background, over-expression, knock-down, knock-out, etc), and neoplasms. The annotation of each experiment and sample was performed manually to provide more detailed information, to detect annotation errors or redundant datasets across published studies. Raw data was quality controlled and subsequently normalized at two levels: 1) intra-experiment normalization using RMA, and 2) inter-experiment normalization using global experiment scaling. This global normalization allows integrating absolute expression values across hundreds of experiments. Therefore, it is possible to calculate average vectors of expression from all samples from the same category. Figure 1 shows the general process of data transformation, from the retrieval from public repositories through data curation to the summarization of expression vectors into representative datasets. While the online tool GENEVESTIGATOR allows scientists to explore the global curated content to identify significant biological effects, selected post-processed reference datasets from GENEVESTIGATOR are made freely available http://www.expressiondata.org.

**Figure 2:**
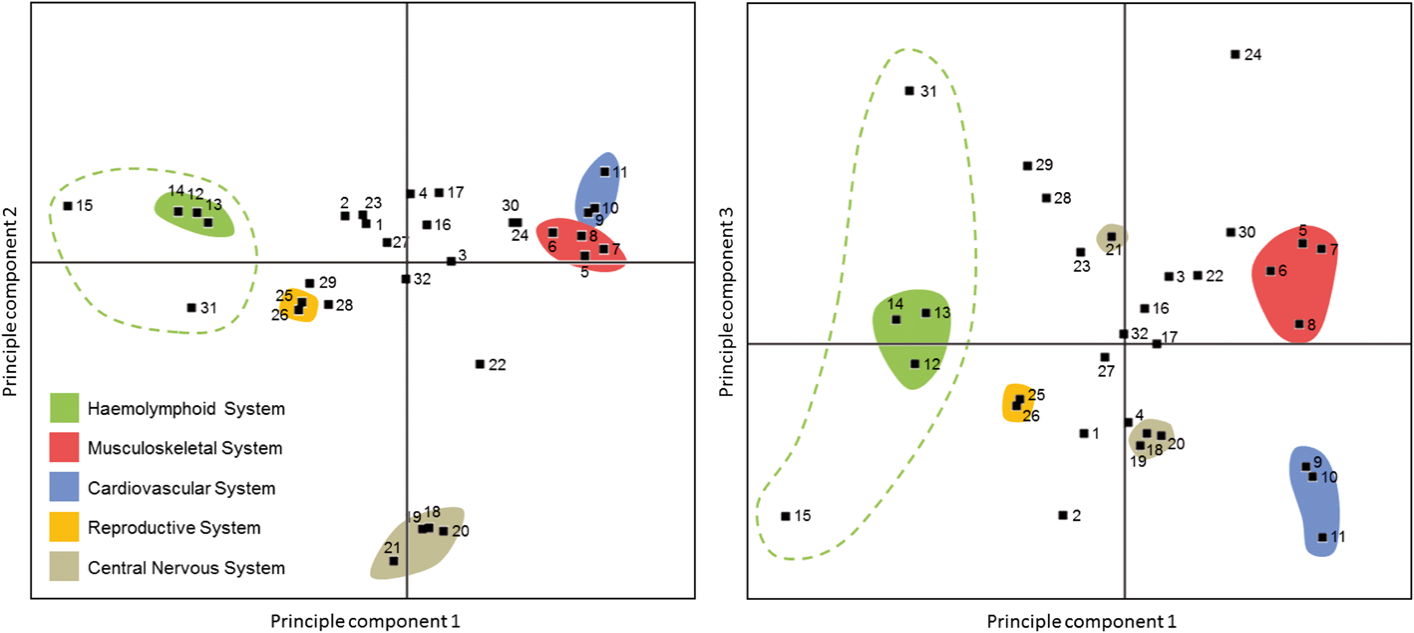
**Principle component analysis of mouse tissues and organs (Left figure: components 1 versus 2; right figure: components 1 versus 3).** Major organ systems are colored, while individual tissues are numbered as follows: (1) cell culture / primary cell, (2) fibroblast, (3) myoblast, (4) adipocyte, (5) hindlimb skeletal muscle, (6) gastrocnemius, (7) muscle skeletal muscle, (8) diaphragm, (9) heart, (10) heart ventricle, (11) heart left ventricle, (12) haemolymphoid system, (13) blood, (14) leukocyte (white blood cell), (15) thymus, (16) integumental system, (17) mammary gland (breast), (18) telencephalon (cerebrum), (19) cerebral cortex (neopallium), (20) hippocampus, (21) cerebellum, (22) eye, (23) intestine, (24) liver, (25) reproductive system, (26) testis (male gonad), (27) lung, (28) endocrine system, (29) pancreas, (30) adipose tissue (fat), (31) bone marrow, (32) mean of all tissues.

The datasets chosen for the ExpressionData resource were typically generated from hundreds of experiments. For each anatomy or development category we calculated a representative vector of expression (meta-profile) for all probe sets from a given microarray platform. For example, all 616 human samples hybridized on the Affymetrix Human133 Plus 2.0 array and which were annotated as “liver” were combined into a single mean expression vector representing the tissue “liver”. We assume that this summarization into meta-profiles generates biologically representative expression vectors. As a proof of concept, we performed a Principle Component Analysis of the anatomy meta-profiles of 31 different mouse tissue types. The projections show a biologically meaningful clustering of related tissue types (Figure 2), even if they were composed of data generated in different laboratories and under different experimental conditions. Almost identical clusters are obtained when clustering the tissue meta-profiles of human or rat data, revealing a high representativeness of the data [15].

The datasets made available at ExpressionData represent a carefully chosen subset of platforms and conditions from the complete GENEVESTIGATOR database. The criteria for selecting a particular condition were defined as follows: 

• Anatomy: each tissue type is represented by data from at least two independent experiments and at least 30 replicates;

• Development: all expression data available for each category is aggregated into an average vector per category.

### Datasets representing spatial expression

The knowledge about the spatial expression characteristics of genes is crucial for understanding their function and regulation. Representative vectors of expression in tissue types were processed from a very large number of samples carried out in at least two independent laboratories under a variety of conditions. Since all datasets are normalized to allow integrating datasets from multiple sources, a large number of samples from different tissues can be compiled into a single data set where each row represents one gene and each column represents one tissue.

To demonstrate the biological validity of tissue meta-profiles, we carried out a principle component analysis of a mouse tissue expression dataset which had been summarized from more than 3000 Affymetrix array datasets available in GENEVESTIGATOR. The results show a clear grouping of tissues that are functionally related (see Figure 2). The first principle component separates distinctly all central nervous system tissues from all other body parts. The second principle component groups all other tissues into clusters of anatomical parts that have a common origin or physiology. For example, a variety of muscle tissues form a distinct cluster that is located close to heart and heart ventricle tissues. These results confirm previous findings on comparing human and mouse tissues based on datasets that were normalized differently and in which tissue samples are represented individually [18]. Differences between the individual vectors therefore primarily reflect fundamental biological processes that are associated with each tissue type. The anatomical datasets available at http://www.expressiondata.org have a carefully selected coverage of tissue types, each of them represented by a single vector of expression.

### Datasets representing developmental expression

**Figure 3:**
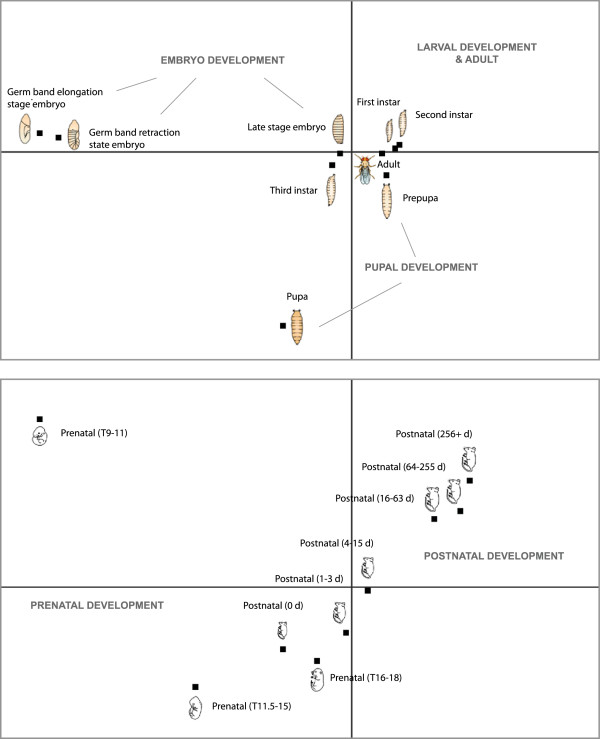
**Principle component analysis of Drosophila and mouse developmental stages.** The expression vector for each stage represents an average from all samples annotated as belonging to that stage of development. Results were processed from data generated with the Affymetrix microarray platforms Drosophila Genome 2.0 (1431 samples) and Murine Genome U74Av2 (2357 samples).

For model organisms such as Drosophila or mouse, datasets covering multiple stages of development represent an interesting source of scientific information. Here, we generated representative expression vectors for each stage of development as the mean of all samples annotated with a given developmental stage ontology category. Each developmental stage is represented by a variety of tissues and conditions occurring at that stage and which are available in the GENEVESTIGATOR database. To illustrate the use of summarized datasets for development, we created meta-profiles for mouse and Drosophila and clustered them using PCA (Figure 3). For mouse, the order by which the stages appear on the projections is chronological, from the oocyte stage up to the adult mouse. The results for Drosophila show three main clusters consisting of a) the instar larvae stages, pre-pupa and adult fly, b) the germ band elongation and retraction stages, and c) the pupa itself. One can distinguish two axes representing embryo and pupal development, respectively. Similarly, mouse development appears to follow two axes, as determined by the two principle components, dividing pre-natal and post-natal processes. The post-natal stages are almost linearly aligned and in the correct biological chronological order, suggesting that expression vectors aggregated from many independent experiments contain biologically representative data.

### Other datasets

Meta-profile data for anatomy and development provide an excellent basis for genomic data interpretation. For many biological questions, however, it is desirable to look beyond the spatio-temporal aspects of gene expression. Many organisms undergo time-related regulation, especially circadian. The ExpressionData resource therefore contains further datasets of particular biological relevance. Two of them are presented here.

### Datasets with biological oscillations

**Figure 4:**
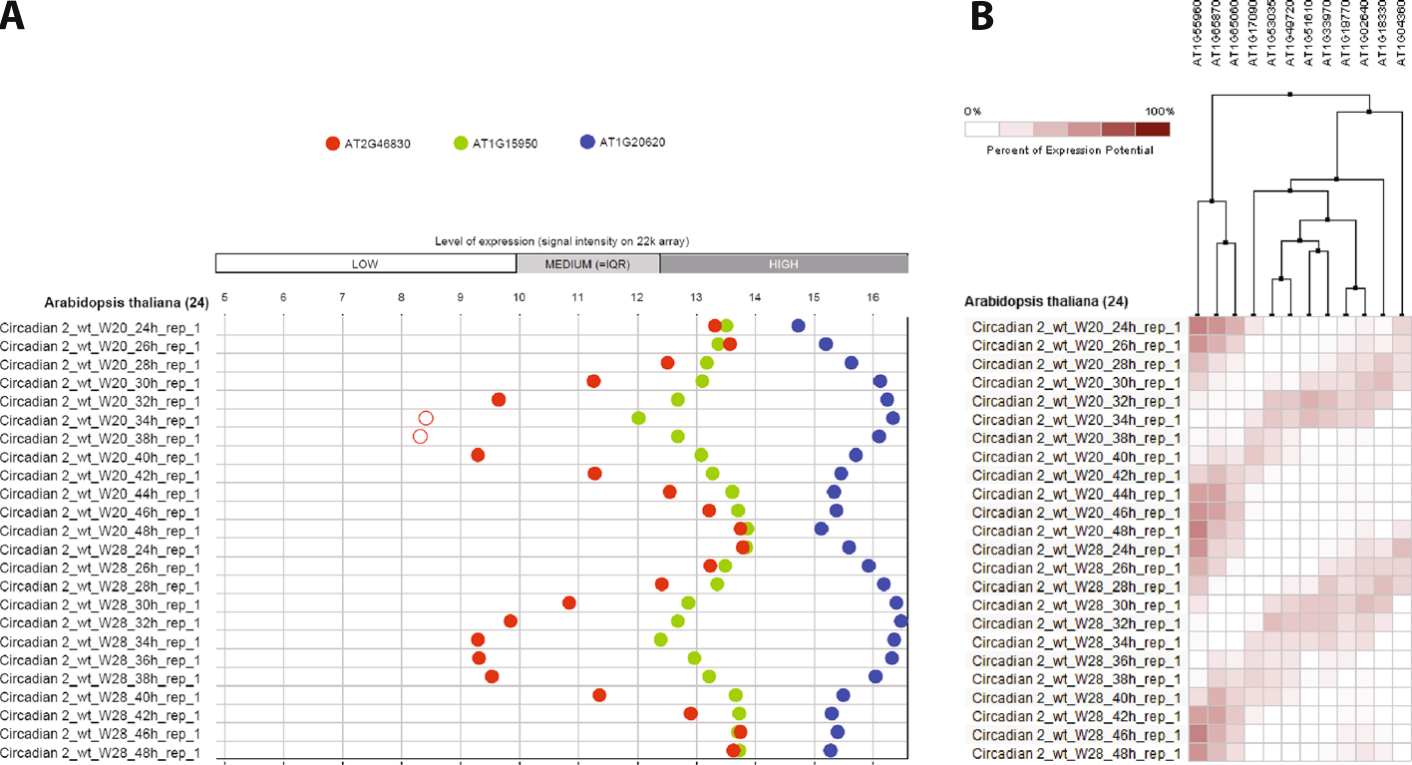
**Oscillatory pattern of expression in Arabidopsis.A)** Three genes with different patterns of oscillation are shown: CCA1 (AT2G46830; circadian clock associated 1; in red), CCR1 (AT1G15950; cinnamoyl coa reductase 1; in green), and CAT3 (AT1G20620; Catalase 3, in blue). Signal values represent expression levels as normalized using RMA. Open circles represent expression not significantly above background (p > 0.06, as obtained from the MAS5 p-values for detection calls). **B)** Twelve genes with different phase shifts in the circadian oscillation pattern relative to the first gene. Genes were clustered in GENEVESTIGATOR using Hierarchical Clustering with optimal leaf ordering.

Many biological processes are repetitive or timed, leading to oscillations in their regulation. Two typical examples of oscillatory behaviour are circadian rythms and the cell cycle. In ExpressionData, several public experiments having at least one complete oscillation were curated and are made available. For example, the Arabidopsis circadian clock experiment available at NASC under experiment ID NASCARRAYS-196 (http://www.ncbi.nlm.nih.gov/geo/query/acc.cgi?acc=GSE5612) is a series of Arabidopsis samples collected at 2 hours interval over a period of 48 hours and across two different schemes of day/night entrainment. As an example, Figure 4 shows three marker genes for circadian regulation across these samples.

### Datasets with time-courses

Time-course datasets are useful to identify trends and to measure the rapidity of response to a given perturbation or developmental process. This type of data is also interesting for the development of methods to identify such trends. Here, we show an example of a time-course dataset that, as opposed to the circadian dataset, was used to identify genes having no circadian response.

The response to cold stress is a highly conserved defense mechanism by which plants protect their viability [19]. The cold stress response can be an attractive mechanism to modulate the expression and production of recombinant protein in plants without usage of chemical inducers, but via relatively inexpensive and controllable stimuli.

The dataset considered in this section was generated by harvesting leaves of 6 week old greenhouse-grown tobacco (*Nicotiana tabacum*) plants from the relatively cold-intolerant Flue-Cured variety K326. Leaves were quickly chilled for 10 minutes in a blast chiller at a temperature between 0-5°C and monitored to avoid frost. Then, the samples were incubated at the same temperature for 5 hours or 24 hours before being frozen in liquid nitrogen. Two harvesting times, one in the morning (7:30am) and one in the afternoon (1:00pm) were chosen in order to avoid circadian effect. Two sets of control leaves were harvested together with the cold-treated samples and frozen immediately in liquid nitrogen (Time 0). Three biological replicates were prepared for each time point. Unlike in the previously described datasets focused on biological oscillations, the objective of this type of studies is to find genes that are continuously induced by cold treatment over the time period of 24 hours independently of harvesting time of the seedlings (i.e. biological oscillation between morning and afternoon do not interfere with the gene inductions). The raw data from this experiment are available in Gene Expression Omnibus (GEO) under accession Nr. GSE44938.

**Figure 5:**
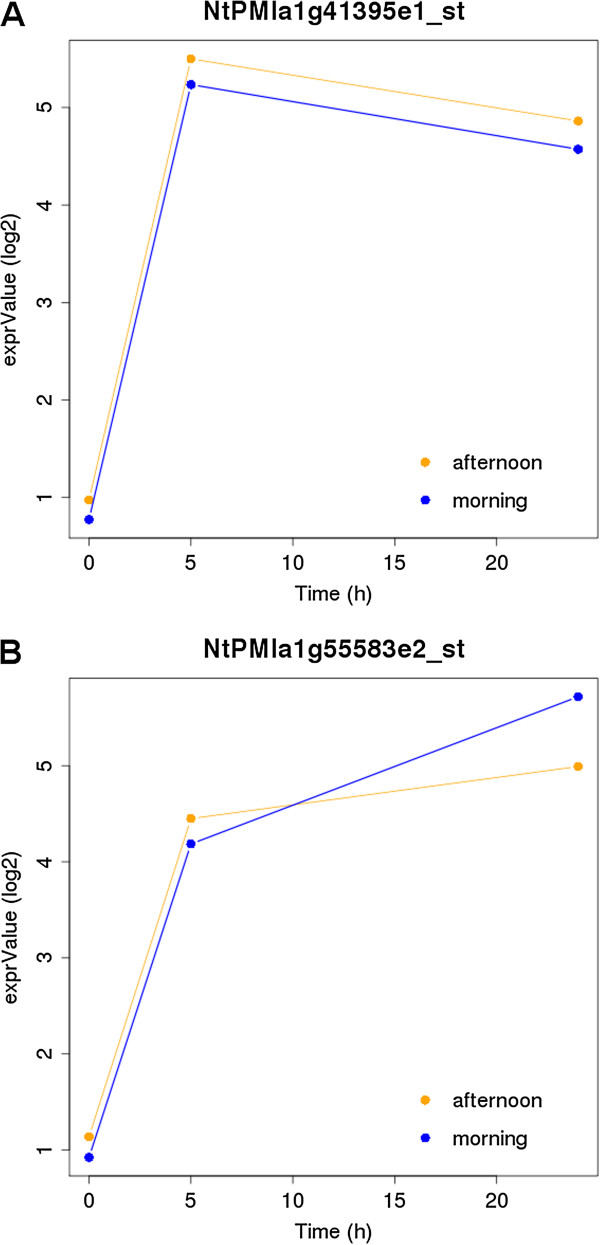
**Non-circadian time course gene expression of two early cold-induced exons.A**. Putative C-repeat-binding factor 4/dehydration-responsive element-binding protein 1D (CBF4/DREB1D) **B**. Putative Ethylene-Responsive element binding ERF2.

Figure 5 shows a typical behavior of two non-circadian early induced genes. Figure 5A describes the cold induction of a gene identified by the Tobacco Exon Array probeset NtPMIa1g41395e1 [20]. This exon is induced 16-fold and annotated as similar to At5g51990, or CBF4/DREB1D (C-repeat-binding factor 4/dehydration-responsive element-binding protein 1D). CBF4 is reported to be a transcription factor key responder to low temperatures [21] in different higher plants (Arabidopsis [22], cereals [23,24], grapevine [25,26]). Figure 5B depicts the dynamics of induction of a gene annotated as Ethylene-Responsive element binding protein ERF2 (probeset numbers NtPMIa1g55583e1_st and NtPMIa1g55583e2_st from Tobacco Exon Array [20]). The expression of the gene achieves 8-fold at 5 hours and 16-fold increase in 24 hours. The ERF family of genes is known to be differentially regulated in Arabidopsis [27], soybean [28], tomato and tobacco [29] by abiotic stress conditions including wounding and cold: the induction of this gene in our experiment in fact is most likely due to the double stimulus wound/cold following harvesting and cold treatment procedures.

Microarray data experiments such as this may lead to the discovery of regulative elements which serve as powerful tools for expression of commercially valuable recombinant proteins in plants, and which are not circadian-sensitive. They may also lead to the identification of new unknown genes which could be targeted for selection of cold tolerant varieties.

## Utility

The datasets presented here are examples of curated and aggregated datasets of gene expression covering a broad range of biological conditions. We have created reference data sets that can be used for 

• Interpreting results from other studies. The created expression compendia, such as for anatomical parts, are representative for individual biological states and allow comparisons with expression data obtained in other experiments.

• Finding novel gene regulatory modules and networks from aggregated datasets containing a high diversity of tissues and developmental stages.

• Testing bioinformatics methods or algorithms with manually curated and biologically relevant data.

The results shown here and on the http://www.expressiondata.org website demonstrate the biological relevance of such datasets. In fact, the results in Figure 2 and examples from the http://www.expressiondata.org website demonstrate that the compiled data contain robust and representative estimates for individual biological states such as tissue types or stages of development. In particular, a high concordance was found in the tissue transcriptomes between human and mouse by comparing aggregated expression data for each organism across the same set of tissues. While the ExpressionData resource is expected to grow as new datasets are processed and made available, it does not aim at having complete sets of data for every organism.

## Conclusions

ExpressionData is a new source of tested, high quality, manually curated, globally normalized and aggregated expression data ready for use in a variety of data interpretation and verification tasks. It is expected to simplify the search for robust and high quality datasets and to provide a set of reference data for comparison. Its content is open access and freely available for download at http://www.expressiondata.org.

## Competing interests

The authors declare that they have no competing interests.

## Authors’ contributions

All authors were involved in data collection, data processing and creating the resource. PZ, SB, OL, NVI, FM and WG wrote the manuscript. All authors read and approved the final manuscript.
